# Long-Axis Rotation of Jaws of Bamboo Sharks (*Chiloscyllium plagiosum*) During Suction Feeding

**DOI:** 10.1093/iob/obac024

**Published:** 2022-07-25

**Authors:** Bradley R Scott, Elizabeth L Brainerd, Cheryl A D Wilga

**Affiliations:** Department of Evolution, Ecology, and Behavior, University of Illinois Urbana-Champaign, 61801 IL, USA; College of the Environment and Life Sciences, University of Rhode Island, Kingston, 02881 RI, USA; Department of Ecology, Evolution, and Organismal Biology, Brown University, 02912 RI, USA; College of the Environment and Life Sciences, University of Rhode Island, Kingston, 02881 RI, USA

## Abstract

Long-axis rotation (LAR) of the jaws may be an important component of vertebrate feeding mechanisms, as it has been hypothesized to occur during prey capture or food processing across diverse vertebrate groups including mammals, ray-finned fishes, and sharks and rays. LAR can affect tooth orientation as well as muscle fiber direction and therefore muscle power during feeding. However, to date only a handful of studies have demonstrated this LAR in vivo. Here, we use XROMM to document LAR of the upper and lower jaws in white-spotted bamboo sharks, *Chiloscyllium plagiosum,* during suction feeding. As the lower jaw begins to depress for suction expansion, both the upper jaw (palatoquadrate) and lower jaw (Meckel's cartilage) evert, such that their toothed surfaces move laterally, and then they invert with jaw closing. Eversion has been shown to tense the dental ligament and erect the teeth in some sharks, but it is not clear how this tooth erection would contribute to suction feeding in bamboo sharks. Two recent XROMM studies have shown LAR of the lower jaws during mastication in mammals and stingrays and our study extends LAR to suction feeding and confirms its presence in shark species. Examples of LAR of the jaws are becoming increasingly widespread across vertebrates with unfused mandibular symphyses. Unfused lower jaws are the plesiomorphic condition for most vertebrate lineages and therefore LAR may be a common component of jaw mechanics unless the mandibular symphysis is fused.

## Introduction

**Fig. 1:**
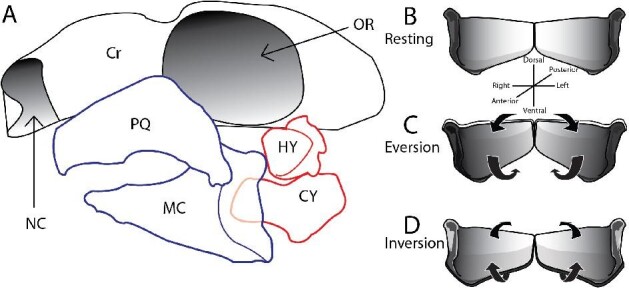
(A) Lateral view of *Chiloscyllium plagiosum* outlining the mandibular arch (blue) and the hyoid arch (red). (B, C, D) Anterior view diagram of hypothesized long-axis rotation of the lower jaw of *C. plagiosum*. (B) Meckel's cartilage in anterior view at rest, (C) during eversion, and (D) during inversion. Cr = cranium, CY = ceratohyal, HY = hyomandibula, MC = Meckel's cartilage, NC = nasal capsule, OR = Orbit, PQ = palatoquadrate.

Long-axis rotation (LAR) of the jaws can modify feeding mechanics by changing the orientation of teeth and the directions of muscle fibers during jaw abduction or adduction. This extra axis of rotation could allow animals to feed on a wide variety of foods or improve feeding performance beyond what is possible without LAR. Distinct from depression or medio-lateral rotation of the upper or lower jaw at the jaw joint, LAR describes the rotation of a skeletal element around its long axis (Fig. 1). During LAR, the dental surface—functional tooth surface, dorsal margin of lower jaw and ventral margin of upper jaw—moves laterally/labially during eversion (Fig. 1C) or medially/lingually during inversion (Fig. 1D).

LAR of vertebrate jaws is hypothesized to be a key factor in the feeding mechanisms of a variety of vertebrates (Brainerd and Camp 2019). However, LAR of the jaws has only been documented in a handful of mammal taxa (Kallen and Gans 1972; Oron and Crompton 1985; Bhullar et al. 2019; Williams 2019), and in freshwater stingrays (*Potamotrygon motoro*, Laurence-Chasen et al. 2019). Jaw LAR has been hypothesized for anteaters (Naples 1999), bats (Kallen and Gans 1972), *Tenrec* (Oron and Crompton 1985), a cichlid fish (Aerts 1985), oceanic sharks (Frazzetta and Prange 1987; Chappell and Seret 2021), and white spotted bamboo sharks (Ramsay and Wilga 2007). Since the advent of XROMM, LAR has also been confirmed for limb bones during locomotion (Kambic et al. 2014; Mayerl et al. 2019) and hyoid elements during feeding (Scott et al. 2019).

Advances in X-ray video recording have improved the reconstruction of three-dimensional kinematics of feeding among vertebrates (Brainerd et al. 2010; Brainerd and Camp 2019; Williams 2019). Recent studies with X-ray Reconstruction of Moving Morphology (XROMM) have revealed additional degrees of motion in the jaws of vertebrates. LAR of the jaws has been documented with XROMM during food processing (Bhullar et al. 2019; Laurence-Chasen et al. 2019), but may be present during a wide range of feeding behaviors. In this study, LAR of the upper and lower jaws (palatoquadrate and Meckel's cartilage in Chondrichthyes) was recorded during suction feeding trials of white spotted bamboo sharks (*Chiloscyllium plagiosum* Bennet 1830) expanding the behavioral and taxonomic breadth of LAR within vertebrates, and strengthening the possibility that LAR is a common component of jaw mechanics among vertebrates.

## Materials and methods

The dataset analyzed for this study was also used for a study of hyoid arch kinematics (Scott et al. 2019), food transport (van Meer et al. 2019), and pectoral girdle kinematics (Camp et al. 2017). Marker implantation, CT scan, and X-ray video data collection procedures were identical to Scott et al. (2019) and are described very briefly below.

White-spotted bamboo sharks (*C.plagiosum* Bennett) were anaesthetized using 0.033 gL^–1^ of MS-222 tricaine methanesulfonate in buffered seawater for marker implantation and CT scan procedures. Four conical tungsten carbide markers (Kambic et al. 2014) were implanted in the chondrocranium, and three markers in each of upper jaw, lower jaw, hyomandibula, ceratohyal from the left side (Fig. S1). Individuals had body lengths of 78.6 cm, 79.2 cm, 85.0 cm, and cranium lengths of 6.47 cm, 6.35 cm, 6.58 cm. All experimental procedures were approved by Institutional Animal Care and Use Committees of Brown University and the University of Rhode Island. All sharks recovered and fed normally following implantation.

Sharks were CT scanned using a FIDEX CT scanner (Animage, Pleasanton CA) at an isotropic resolution of 0.185 mm. Mesh models of the cranium, upper and lower jaws, hyomandibula, and ceratohyal as well as implanted markers were created with Osirix (Pixmeo, Geneva, Switzerland) and exported as .obj files to Maya (2015; Autodesk). The centroid for the vertices of each marker was calculated using the *vAvg* tool in the XROMM MayaTools (bitbucket.org/xromm/xromm_mayatools).

Feeding trials were recorded at the W. M. Keck Foundation XROMM Facility, Brown University (Imaging Systems and Service). Food was withheld for one week before data collection. Individuals were fed one piece of squid or herring for each trial and each piece was cut to roughly 50% of gape width. The motion of implanted markers was recorded at 320 Hz or 330 Hz, with X-ray energies of 110–120kV and 100mA. Video data are stored on xmaportal.org with their essential metadata in accordance with best practices for video data management in organismal biology (Brainerd et al. 2017).

Marker positions were tracked using XMALab (Knorlein et al. 2016) and used to calculate translations and rotations of each element. The jaws are composed of tessellated cartilage, a very stiff form of cartilage (Seidel et al. 2021). The jaws of the bamboo sharks can therefore be assumed to act as rigid bodies, a prerequisite for motion tracking in XROMM (Brainerd et al. 2010). Rigid body transformations were imported into Maya and applied to 3D meshes of each element imported from the CT scans (XROMM MayaTools, *imp*). The mean standard deviations of marker-to-marker distance pairs in each rigid body were calculated to estimate the precision of marker tracking (Brainerd et al. 2010; Knörlein *et al.* 2016). A grand mean s.d. of 0.12 ± 0.016 mm was based on a total of 60 s.d. from five rigid bodies per individual measured for one trial for each individual (1800–2200 frames per trial).

### Joint coordinate systems

Rotations of each element in each trial were calculated using joint coordinate systems (JCSs). In this study, we measured the difference in motion between the lower jaw and the chondrocranium, and between the upper jaw and the chondrocranium. JCS data was output as Euler angles using *jAx* in XROMM MayaTools. Rotation order was set as z-y-x. The z-axis measured the largest expected rotation, depression–elevation for the upper and the lower jaws. Depression was negative, elevation positive. The x-axis was aligned along the long axes of the upper jaw and lower jaw to capture LAR. Positive rotations are inversions (tooth row rotates medially) and negative rotations are eversions (tooth row rotates laterally, Bhullar et al. 2019). The x-axis points in the cranial direction for the lower jaw and caudal direction for the upper jaw to maintain the meaning of inversion and eversion. The y-axis captured rotations of the jaws that moved the jaw joint medially (negative for lower jaw and positive for upper jaw) and laterally (positive for lower jaw and negative for upper jaw). Timing of peak rotation was recorded in milliseconds relative to maximum gape and magnitude of rotation relative to the chondrocranium was recorded in degrees of rotation around each axis of the upper and lower jaws. A total of 12 trials were analyzed for three individuals; four trials per individual.

## Results

### Feeding sequence

Food was captured by inertial suction and transported to the oral cavity in a single motion for all trials analyzed except one. For the single exception the food item was drawn to the edge of the mouth, grasped, and then transported in another suction event. While the magnitude of hyoid motions of that trial was lower than other trials (Scott et al. 2019) no obvious effect on the mandibular arch motions was found. Cranial elevation was present prior to feeding in some trials to position the head in front of the food; however, little or no cranial elevation was observed during feeding strikes, making the cranium a reliable base for estimating relative motions of the jaws. Due to the increased accuracy of XROMM, opening of the mouth can be recorded earlier relative to peak gape compared to other kinematic methods used to study feeding in bamboo sharks (Wilga 2008; Wilga et al. 2012). Initiation of the expansive phase is typically set as when the gape reaches 10% of maximum gape for that feeding event to exclude ventilatory motions (Wilga and Sanford 2008); however, some motions, such as eversion of the lower jaw, begin prior to 10% of maximum gape. Here, initial jaw depression is defined as the frame after the last ventilatory jaw closure before maximum gape. Timing of motions are recorded relative to peak gape (set as Time = 0 ms) to compensate for the high variation in timing of events caused by gape opening for ventilation prior to feeding.

**Fig. 2:**
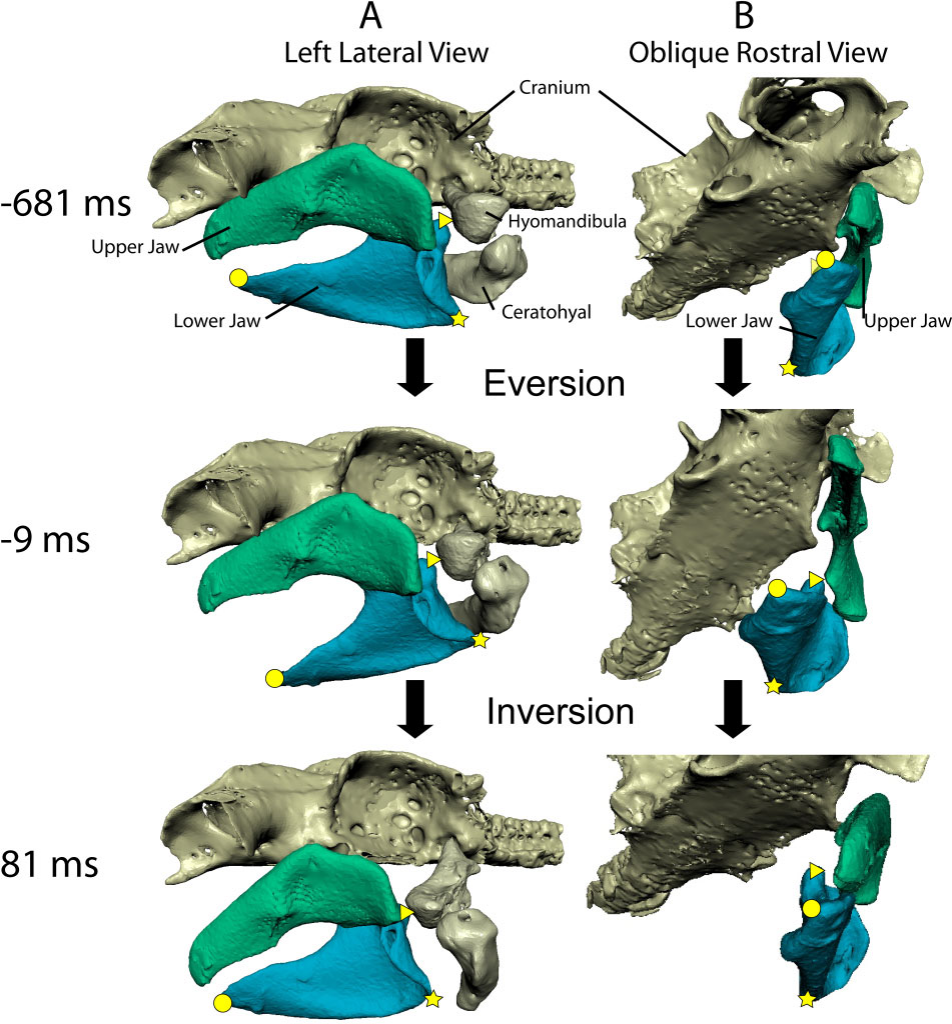
Opening, closing, and long-axis rotations of the jaws during a sequence of feeding for Bamboo 03 in (A) lateral view and in (B) ventral-oblique view rostral to lower jaw with hyoid elements removed. Cartilages are shown at resting position (−681 ms), maximum eversion (−9 ms), and maximum inversion (81 ms) of the lower jaw. All times relative to maximum gape. The lower jaw (Meckel's cartilage) is light blue and the upper jaw (palatoquadrate) is green. Shapes denoting landmarks are given for the lower jaw to demonstrate changes in orientation along the long-axis: triangle, mandibular knob; star, mandibular angle; circle, distal tip of the lower jaw.

Feeding was initiated with simultaneous depression and eversion of the lower jaw (Fig. 2A at −681 ms, Fig. 2B at −9 ms), causing the dental surface of lower jaw to move laterally. Near peak gape the jaw joint began to protrude, moving rostrally, as well as ventrally and medially (Fig. 2A at −9 ms). The upper jaw also began to evert prior to peak gape, rotating the dental surface laterally. Maximum protrusion of the jaw joint coincided with the onset of depression and retraction of the ceratohyal and adduction and protrusion of the distal end of the hyomandibula (Fig. 2A, −9– 0 ms). Peak depression of lower jaw did not always coincide with peak gape, as variation in the onset of upper jaw depression among trials was high. At, or slightly before, peak gape, inversion of the lower jaw began and eversion of the upper jaw peaked while the distal end of the upper jaw began to protrude rostrally and ventrally (Fig. 2, 81 ms). At jaw closure, the lower jaw was fully inverted with the jaw joint maximally protruded and constricted medially (Fig. 2, 81 ms). During recovery, the lower jaw and the upper jaw retracted and elevated towards the original position, and often began physical breakdown (processing) of the food prior to full recovery.

### Rotations from joint coordinate systems (JCSs)

**Fig. 3:**
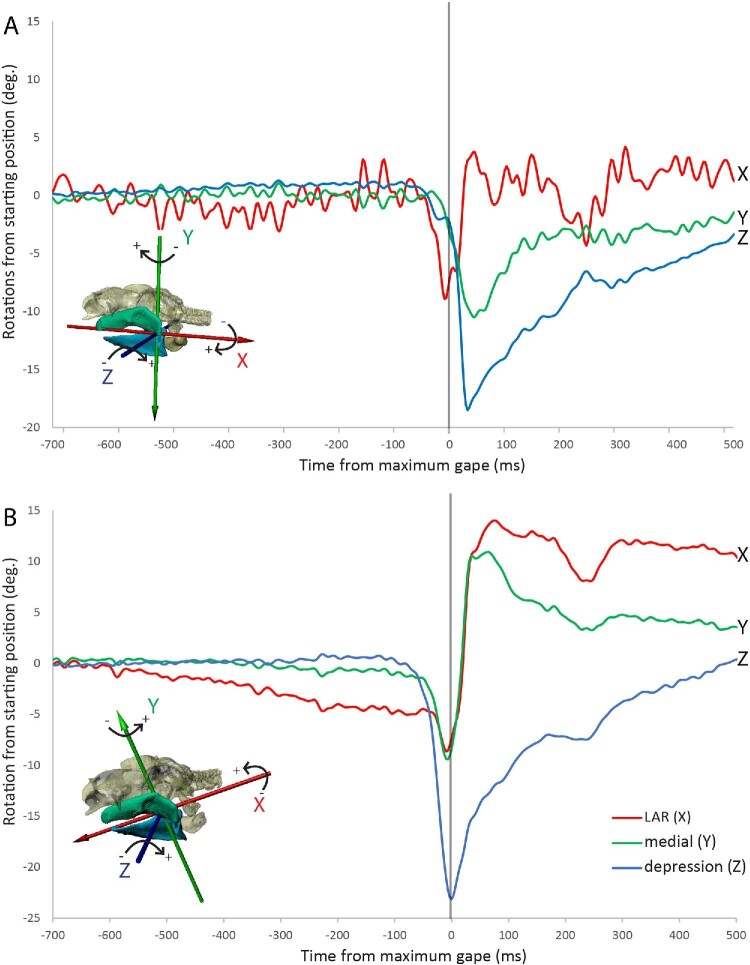
Graphs of rotations of (A) upper jaw and (B) lower jaw relative to the cranium for a single trial of Bamboo 03. Rotations were measured along three axes (X, Y, Z, shown in inset) using Joint Coordinate Systems aligned with the upper and lower jaws. The long-axis rotation (LAR) plot is noisy for the upper jaw because markers had to be placed close to the x-axis, thus amplifying the effect of marker tracking noise for that degree of freedom.

LAR of the upper and the lower jaws can be interpreted as slight medial and lateral displacements of the ventral margins relative to the jaw joint (lower jaw) or cranium (upper jaw) in anterior views (Fig. 2B) and are best quantified using JCSs (Fig. 3). Eversion of the lower jaw is a smaller motion than inversion, with means (± s.e.m.) of −8.0 ± 1.1 deg and 11.2 ± 1.5 deg from starting position, respectively (Fig. 3A). Eversion of the upper jaw (12.3 ± 0.9 deg) is slightly greater than inversion of the lower jaw (11.2 ± 1.5 deg). Depression of the lower jaw is the largest rotation of the jaw joints at −22.7 ± 0.6 deg., although depression of the upper jaw is also moderately high at −15.7 ± 1.8 deg (Fig. 3). Depression of the upper jaw can be interpreted as ventral protrusion of the jaw during feeding (Wilga and Sanford 2008; Scott et al. 2019).

Motions of the mandibular arch are more sequential compared to the hyoid arch, where the majority of the motion happens at the same time (Scott et al. 2019). Maximum eversion of the lower jaw at −19 ± 7 ms is followed by maximum eversion of the upper jaw at 0 ± 1.6 ms, then depression of the lower jaw at 6 ± 2 ms, depression of the upper jaw at 55 ± 5 ms and finally maximum inversion of the lower jaw at 87 ± 6 ms after peak gape (Fig. 3).

## Discussion

When LAR of the jaws is recorded in vivo it is associated with unfused symphyses of the jaws (e.g., Lieberman and Crompton 2000; Bhullar et al. 2019; Laurence-Chasen et al. 2019; Williams 2019). This association is limited in sample size, but consistent across a wide range of taxa and whereas prior studies measured LAR in vivo during food processing behaviors, this study extends the behavioral breadth of LAR to suction feeding. Fusion of the mandibular symphysis is a derived trait when it occurs so the plesiomorphic condition is an unfused symphysis in most major vertebrate lineages including mammals (Scapino 1981; Lieberman and Crompton 2000), squamates (Holliday et al. 2010), archosaurs (Holliday and Nesbitt 2013), chondrichthyans, and actinopterygians (Carroll 1988), and among the earliest jawed vertebrates (Zhu et al. 2013; Li et al. 2021). If jaw LAR is widespread among vertebrates with unfused jaw symphyses, then LAR may be a significant component of the jaw mechanics of most vertebrates even though many current models of jaw mechanics across vertebrates do not include LAR (e.g., Lauder 1979; Muller et al. 1982; Westneat 2003, 2004; Thompson et al. 2003; Curtis et al. 2010; Coates et al. 2019).

### Modeling LAR

The impact of LAR on force production or muscle orientation has not been considered in models of feeding. For models of speed and force, LAR has the potential to alter the orientation of muscle fibers during feeding as well as the application of stress and strain. For example, eversion can lengthen muscle fibers. Longer fibers increase jaw-closing forces and fiber length can be a significant component of bite force models (e.g., Ferrara et al. 2011). Future models of feeding mechanics among vertebrates should consider whether LAR of the jaws is possible and, if so, what impact it could have on feeding mechanics. So far, the wide taxonomic and behavioral breadth of jaw LAR precludes clear anatomical correlates for this degree of freedom. LAR can be excluded when the jaws are either fixed to the skull (e.g., the maxilla in mammals and most tetrapods), or fixed at the symphysis of the jaws through sutures (e.g., primates, ungulates). In all other cases LAR should be considered viable until excluded by kinematic analyses.

### Possible functions of jaw LAR in bamboo sharks

The biomechanical functions, if any, of LAR during suction feeding are unclear. Food is captured at peak gape (van Meer et al. 2019) and white-spotted bamboo sharks evert lower and upper jaws prior to peak gape and then invert the lower and the upper jaws at peak gape (Figs. 2, 3). Eversion of the lower jaw and the upper jaw were predicted previously (Frazzetta and Prange 1987; Ramsay and Wilga 2007; Chappell and Seret 2021) for *C. plagiosum* and large oceanic shark species to tense the dental ligament, erecting the teeth during prey capture. The pattern of LAR we observed is consistent with tensing the dental ligament in the upper jaw during prey capture because eversion occurs during food capture (Ramsay and Wilga 2007), but LAR of the lower jaw is varied and eversion, hypothesized to erect the teeth, may precede prey capture (contra Ramsay and Wilga 2007). For this study food items were kept small to facilitate multiple feedings before the shark was satiated, so food was captured without grasping and therefore may not have induced typical tooth erection behavior; however, erection of the teeth prior to food capture could allow the teeth to puncture into the food drawing it inward as the mouth closes, preventing escape. Notably, there was one trial during which food was grasped; this trial did not have any clear differences in jaw kinematics, including LAR, compared to other trials. Eversion happened despite the sharks not grasping food and so LAR may not be related to, or exclusive to, erecting the teeth. Although we can confirm the presence of LAR of the lower and upper jaws in *C.plagiosum* during suction feeding, we are unable to confirm whether this action tenses the dental ligament and/or erects teeth as predicted (Frazzetta and Prange 1987; Ramsay and Wilga 2007) because no record of tooth motion was possible with our current methods.

Alternatively, LAR could improve suction performance. Eversion of the upper and the lower jaws can make the transverse section of the oropharyngeal cavity more circular during feeding. Circular cross-sections produce less drag, increasing performance by increasing flow speed (e.g., Muller and Osse 1984). Hypothetically, suction performance would be greater when the jaws are everted than if there were no LAR, and LAR could represent an additional component of the bamboo shark suction mechanism. Bamboo sharks can produce among the lowest subambient suction pressures in vertebrates, albeit with a significant amount of variation in performance and kinematics (Wilga and Sanford 2008; Ramsay and Wilga 2017). LAR during suction feeding in ray-finned fishes has also been hypothesized, with the angulo-articular inverting relative to the dentary during mouth opening and potentially deforming the remnant of Meckel's cartilage in torsion (Aerts 1985).

## Conclusions

White spotted bamboo sharks demonstrate LAR of the upper and the lower jaws during suction feeding. Examples of LAR of the jaws are becoming increasingly widespread across vertebrates and across feeding strategies. Recent kinematic analyses using XROMM have found LAR during food processing (Bhullar et al. 2019; Laurence-Chasen et al. 2019; Williams 2019), and this study extends LAR of the jaws to suction-feeding bamboo sharks (*C. plagiosum*) with strong eversion and inversion of the lower jaws and eversion of the upper jaws during food capture. Jaw LAR has also been hypothesized for biting, grasping, and ant eating, based on specimen manipulations and modeling (Oron and Crompton 1985; Frazzetta and Prange 1987; Naples 1999) and could be present in any taxon when contralateral jaw elements are unfused. Given the taxonomic and behavioral breadth of jaw LAR in vertebrates, LAR should be considered an additional range of motion in the feeding mechanics of any vertebrate unless there are anatomical features to prevent it, or kinematic evidence to refute its presence.

## Data Availability

All raw and processed XROMM data for this study are available on xmaportal.org, study identifier URI1, in Public Collection, Bamboo shark suction feeding trials https://xmaportal.org/webportal/larequest.php?request=CollectionView&StudyID=1&instit=URI&collectionID=1.
